# Prion therapeutics: Lessons from the past

**DOI:** 10.1080/19336896.2022.2153551

**Published:** 2022-12-14

**Authors:** Kyu Hwan Shim, Niti Sharma, Seong Soo A An

**Affiliations:** Department of Bionano Technology, Gachon University, Seongnam, South Korea

**Keywords:** Prions disease, transmissible spongiform encephalopathies, CJD, anti-prion agents, mode of action, therapeutics

## Abstract

Prion diseases are a group of incurable zoonotic neurodegenerative diseases (NDDs) in humans and other animals caused by the prion proteins. The abnormal folding and aggregation of the soluble cellular prion proteins (PrP^C^) into scrapie isoform (PrP^Sc^) in the Central nervous system (CNS) resulted in brain damage and other neurological symptoms. Different therapeutic approaches, including stalling PrP^C^ to PrP^Sc^ conversion, increasing PrP^Sc^ removal, and PrP^C^ stabilization, for which a spectrum of compounds, ranging from organic compounds to antibodies, have been explored. Additionally, a non-PrP targeted drug strategy using serpin inhibitors has been discussed. Despite numerous scaffolds being screened for anti-prion activity *in vitro*, only a few were effective *in vivo* and unfortunately, almost none of them proved effective in the clinical studies, most likely due to toxicity and lack of permeability. Recently, encouraging results from a prion-protein monoclonal antibody, PRN100, were presented in the first human trial on CJD patients, which gives a hope for better future for the discovery of other new molecules to treat prion diseases. In this comprehensive review, we have re-visited the history and discussed various classes of anti-prion agents, their structure, mode of action, and toxicity. Understanding pathogenesis would be vital for developing future treatments for prion diseases. Based on the outcomes of existing therapies, new anti-prion agents could be identified/synthesized/designed with reduced toxicity and increased bioavailability, which could probably be effective in treating prion diseases.

## Introduction

Prion diseases (Transmissible spongiform encephalopathies: TSEs) are a set of rare and deadly zoonotic neurodegenerative diseases (NDDs), affecting both humans (Creutzfeldt-Jackob disease: CJD, variant CJD: vCJD, Fatal familial insomnia: FFI, Gerstmann-Straussler-Scheinker syndrome: GSS, Kuru) and animals (Bovine spongiform encephalopathy: BSE, Chronic wasting disease: CWD, Scrapie, Feline spongiform encephalopathy: FSE, Transmissible mink encephalopathy: TME, Ungulate spongiform encephalopathy: USE).

PrP^C^ is a 32 kDa glycophosphatidylinositol (GPI)-anchored glycoprotein expressed in many tissues and abundantly in the nervous tissues [1]. The molecular pathogenesis of prion diseases included the collection of misfolded prion protein (PrP^Sc^) [2]. The normal PrP (PrP^C^) exists as a monomeric, α-helical, soluble, and protease-sensitive protein [3]. On the contrary, scrapie isoform (PrP^Sc^) is a multimeric, β-sheet rich [4], insoluble, protease-resistant [5,6], and infectious form [7]. The progressive abnormal protein aggregation in the Central nervous system (CNS) affects normal brain structure (formation of spongiform, like holes in the brain tissue) with reduced functions (cognition, memory, movement).

Even though CNS is the key target of prion pathology nevertheless many prion diseases are complemented by prion replication at extra cerebral sites (viz. secondary lymphoid organs, blood, muscle) [8]. PrP^C^ is the responsible molecule for transport via the peripheral nervous system (PNS) into the brain. However, the transport mechanism of neuronal prion from the PNS to the CNS and/or vice versa is poorly understood. Two hypotheses were proposed for prion propagation: the ‘Domino hypothesis’ (prion propagation occurring on the neuronal cell) [9] and the ‘Streetcar hypothesis’ [10] where the nerve endings take up PrP^Sc^ and transport them back to the CNS.

Prion diseases can be acquired (from contaminated foods or medical procedures), sporadic (unknown cause), or genetic (from mutations in the PrP gene: *PRNP*). Truncated mutation of the PrP results in PrP systemic amyloidosis characterized by late-onset sensory and autonomic neuropathy. The symptoms of this disease are comparable to an autonomic dominant NDD, familial amyloid polyneuropathy (FAP), which results from mutations in the transthyretin (TTR) gene [11]. The two diseases can be clinically differentiated by the length of the disease history, FAP being rapidly progressing compared to PrP systemic amyloidosis [12].

Approximately 1–2 people per million are annually affected by prion diseases [13], out of which 85–90% of cases are of sporadic CJD (sCJD) [14]. The major common clinical symptoms of CJD patients include prompt and progressive cognitive debility, difficulty in balance, lack of coordination, visual impairment, and behavioural changes. Some of these symptoms are similar to AD and HD however, CJD patients have a characteristic sponge-like appearance of the brain tissue. The average survival time and survival rate of patients with sCJD in China and other Western countries are 7.1 months with 78.5% mortality within a year of the disease onset [15]. On the other hand, the survival statistics are better in Japan, where the survival period is at the highest of 17.4 months with 46% mortality [16]. The diagnostic tests for CJD are; the detection of periodic synchronous discharge (PSD) or periodic sharp wave complex (PSWC) in electroencephalography (EEG) [17–19], cerebrospinal fluid (CSF) analysis [20], including real-time quaking-induced conversion (RT-QuIC) assays of CSF and nasal-brushing [21,22], cranial magnetic resonance imaging (MRI) [23] and immunohistochemistry of prions in brain tissues from the biopsy.

Numerous reports on prospective targets for treating the prion disease have been investigated. An ample amount of literature is available from various research methodologies, like cell therapy, immunotherapy, pharmacotherapy, and compounds ranging from chemicals to proteins. Compounds destabilizing PrP^Sc^ and reducing infection have also been identified. Many of them revealed encouraging results *in vitro*, while a few *in vivo* studies were performed without success in the clinical trials. As a result, unfortunately, no conclusive treatment for prion diseases existed, hence, only medicines for easing the disease-associated symptoms are being used. For example, antidepressants and sedatives are prescribed for psychological symptoms; myoclonus can be treated with clonazepam and sodium valproate; donepezil, galantamine, rivastigmine, and memantine are used to treat dementia in CJD patients [24,25]. Recently, a prion protein monoclonal antibody (PRN100) has shown promising results in a clinical trial study on CJD patients [14].

The present review provided an updated and comprehensive outlook on various classes of anti-prion agents, the mode of action, and some other important aspects that would be helpful for further research in this field.

## Anti-Prion agents in therapeutics

Nuclear magnetic resonance (NMR) and X-ray crystallography of the PrP^C^ domain revealed a small β-sheet content and α-helical conformation at the C-terminal (amino acid residues 128–231) [26] whereas, the N-terminal region (amino acid residues 23–127) lack proper folding [27] and it is composed octapeptide repeats (OR) (residues 60–91), each repeat with histidine and tryptophan which coordinate up to four copper (Cu II). The fifth copper binding site is located in the non-OR region (His 92 and His111) [28,29]. A palindromic motif (AGAAAAGA) exists between residues 113–120 which is believed to be responsible for prion conversion [30]. The closeness of the palindromic motif to the non-OR region indicates a link between Cu(II) binding and prion conversion [31]. Giachin et al. proposed a model in which PrP^C^ coordinating copper with one His more prone to conversion at acidic conditions suggesting the non-OR region is a key regulator of prion conversion [32]. A recent study used biophysical techniques to demonstrate that the binding of Cu(II) to non-OR region result in compacted conformation and hence affect the structural plasticity of the region. Additionally, Cu coordinate geometries identified as: Type I (closed) and Type 2 (open) for PrP^C^ and PrP^Sc^, respectively [33].

In the pathogenesis of prion diseases, PrP^Sc^ proliferates by seeding its altered conformation in the presence of PrP^C^ as a substrate [34]. Thus, besides acting as a substrate, PrP^C^ oligomers are transducers of neurotoxicity [35]. Hence, the anti-prion agents that can restrict the conversion from PrP^C^ to PrP^C^ oligomers or PrP^Sc^ will be of therapeutic importance. These interactions or polymerization inhibitions can be achieved by binding to PrP^C^ and/or PrP^Sc^. As the plasma membrane is the primary site for the conversion of PrP^C^ to PrP^Sc^ [36, 37

**Figure 1.**
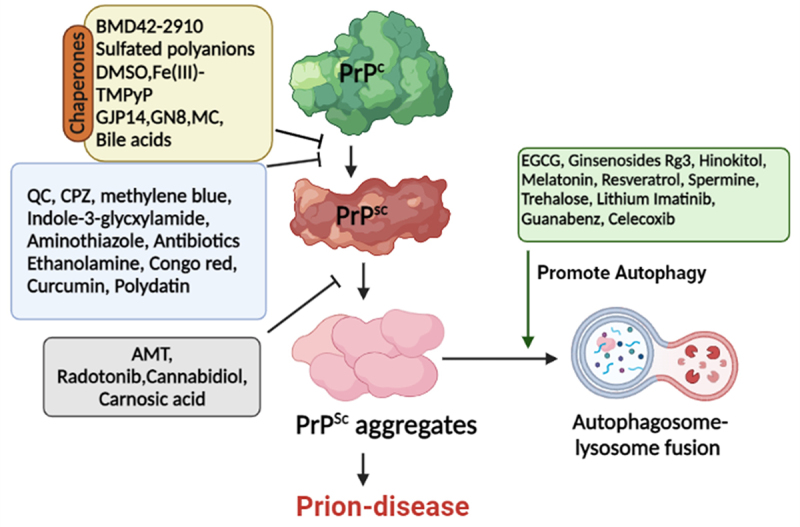
**Schematic representation of some anti-prion agents targeting prion proteins**. PrP^C^, PrP^Sc^, and the conversion of PrP^C^ to PrP^Sc^ are the main targets of these anti-prion agents. Chaperones directly interact with PrP^C^ thereby stabilizing it and preventing/reducing its conversion to PrP^Sc^. Another set of compounds clears the toxic aggregates by promoting autophagy. Abbreviations: PrP^C^: normal prion protein; PrP^Sc^: scrapie prion protein; EGCG: Epigallocatechin-3-gallate; AMT: Aminothiazoles; CPZ: Chlorpromazine; DMSO: Dimethylsulfoxide; QC: Quinacrine.(Prepared by Biorender.com).

], PrP^C^ redistribution is another key target of anti-prion agents. Additionally, subduing PrP^C^ expressions and targeting other pathways to reduce disease-associated neurotoxicity have also been explored. Henceforward, the anti-prion agents work by either of these mechanisms i.e. specific conformational stabilization of PrP^C^; non-specific stabilization of PrP^C^; prevention of PrP^Sc^ accumulation, and preventing proliferation by the interaction of PrP^Sc^ with molecules other than PrP^C^ [38]. Other approaches include the autophagy regulations and the use of chaperones to assist cellular trafficking of PrP^Sc^ and stabilization of PrP^C^, respectively. The mode of action of several anti-prion agents is illustrated in Figure 1.

## 1. Chemical compounds

In this section, various important scaffolds used for anti-prion studies have been discussed. Information on the binding of these compounds to prion protein and the structure-activity relation (SAR) of synthetic derivatives has also been mentioned. Table 1 summarizes various aspects covered in this section.

**Table 1.** Chemical-based anti-prion compounds.

Compound	Class	Structure	Administration route	Target	MOA	Toxicity/BBB penetration	Status	Ref
Anle138b	Diphenylpyrazole derivatives	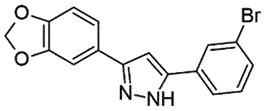	Oral	PrP^C^	Inhibit PrP^Sc^ oligomerization	Safe up to 300 mg/day	Phase 1b study PD patients ongoing	[79–83]
BMD42-2910	Benzoxazole derivative	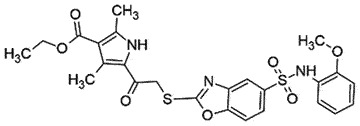	*i.c.*	PrP^C^	Chaperone Reduce PrP^Sc^ accumulation	Non-toxic up to 100 mg/Kg /day in mice	*in vivo*	[45,46]
Congo red	Diazo dye	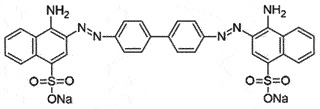	Subcutaneous, *i.p.*	PrP^C^/PrP^Sc^	Block PrP^Sc^ generation	Toxic Poor BBB permeation Non-specific binding to PrP^C^	*In vitro, In vivo* (early)	[59–72]
Cyclodextran	Sulphated polyanions	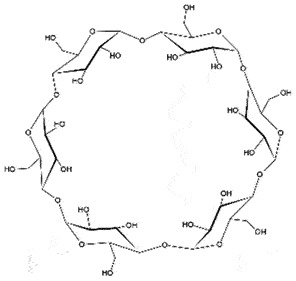		PrP^C^	Chaperone Inhibit PrP^Sc^ Oligomerization	Non-toxic effects (5 mg/kg BW per day)	*In vitro*	[129,130]
Dextran sulphate	Sulphated polyanions	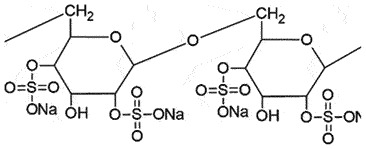		PrP^C^	Inhibit PrP^Sc^ attachment to the membrane and its propagation	NA	*in vitro, in vivo* (early), *ex-vivo*	[123–127]
Dimethyl Sulphoxide	Aprotic solvent	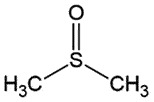	Oral	PrP^C^	Chaperone; Reduce PrP^Sc^ accumulation	Non-toxic up to 0.25 g /day in hamsters	*in vivo*	[84–88]
Ethanolamine	Small organic molecule	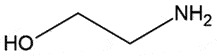			Reduce PrP^Sc^ accumulation		*in vitro*	[89–92]
Fe(III)-TMPyP	Cyclic Tetrapyrroles	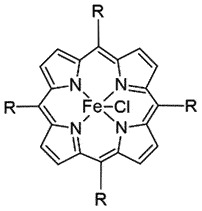	*i.p.*	PrP^C^	Chaperone; Stabilize PrP^C^ Inhibit PrP^Sc^ oligomerization	Poor BBB permeation non-specific interactions with plasma proteins	*in vitro, in vivo*	[49–58]
GJP14	Carbazole derivative	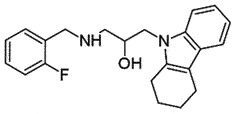		PrP^C^	Chaperone; inhibitor of PrP^Sc^ accumulation		*in vitro*	[47,48]
GN8	Diphenylmethane derivatives	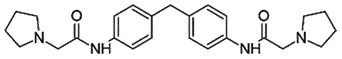	Subcutaneous	PrP^C^	Chaperone Block PrP^Sc^ generation	Non-toxic BBB permeation	Ready for clinical trials	[46,47,73–76]
IND24 IND81 IND125	2-Aminothiazoles	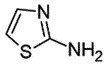	Oral	PrP^Sc^	Inhibit PrP^Sc^ formation	Non-toxic BBB permeation	Failed *in vivo* due to development of drug resistance	[39–44]
Indole-3-Glyoxylamides	Indole-3-Glyoxylamides derivatives	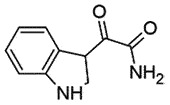		PrP^C^/PrP^Sc^	Inhibit PrP^Sc^ accumulation	No toxicity in Zebrafish	*in vitro*	[97–99]
Lithium & (Li-NP03)	microemulsion		rectal-per-mucosal	PrP^Sc^	Autophagy Reduction in PrP^Sc^		*in vitro; in vivo*	[100–106]
MC	GN8 derivative	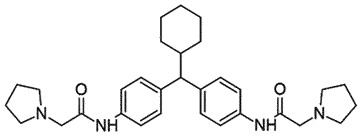	*i.p.*		Chaperone Block PrP^Sc^ generation	No toxicity in animal models upto 30 mg/kg	*in vitro; in vivo; ex-vivo*	[66]
Methylene Blue	Phenothiazine derivative	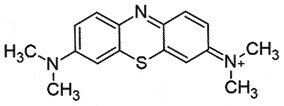		PrP^C^	Block PrP^Sc^ generation	Safe <2 mg/kg BBB permeation non-specific interactions with plasma proteins	*in vitro*	[107,108]
Pentosan Polysulfate	Sulphated polyanions	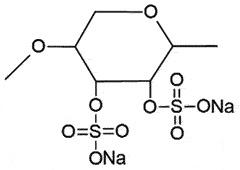	*i.v*. *i.p*. Subcutaneous oral	PrP^C^	Chaperone Stabilize Prp^C^ Downregulate PrP^C^ expression Encourage PrP^C^ internalization and redistribution into late endosomes and/or lysosomes	well-tolerated over a wide dose range (11–110 µg/kg/day) for 6 months Poor BBB permeation	Not effective in clinical trial	[110–122]

**Abbreviations**: *i.v*.: intravascular; *i.p*.:intraperitoneal; *i.c*. intracranial

## 2-Aminothiazoles (2-AMT)

2-AMTs are a class of small molecules with anti-prion activity. Initially, it was proposed that 2-AMTs do not affect PrP^C^ expression, but they can influence PrP^Sc^ formation/removal [39,40]. The improved 2-AMT analogues displayed an effective anti-prion activity (EC_50_ 81 nM) in scrapie’s infected neuroblastoma cells (ScN2a-cl3) through oral administration in the animal model. It readily attained therapeutic steady-state concentration in the brain [41] indicating its ability to cross the blood-brain-barrier (BBB) easily. 2-AMT derivatives, IND24 and IND81, considerably extended the life span of scrapie’s infected mouse or with CWD prion models. However, the mice exhibited advanced neurologic dysfunction in due course, showing the limited efficacy of these 2-AMTs [42]. Another 2-AMT derivative, IND125, can prevent both PrP^Sc^ accumulation and astrocytic gliosis in mice cerebrum, but the progressive CNS dysfunction could not be prevented, probably due to the build-up of PrP^Sc^ in the brain stem [43]. Despite good *in vitro* profile, bioavailability, and extension of survival time in mice, 2-AMTs failed in other animal experiments. Furthermore, combination therapy with IND24 and Anle138b had no significant effect in extending the infection time than monotherapy, proposing the inability of a combination therapeutic approach [44] in the treatment of prion diseases.

## Benzoxazole derivative

BMD42-2910 is a recently discovered anti-prion agent, which exhibited low EC_50_ *in vitro* without toxic effects in the mouse model [45,46]. The compound significantly reduced PrP^Sc^ in prion-infected mice brains and extended the survival time. Molecular docking experiments predicted the binding interactions between PrP^C^ and BMD42-2910 at 4 amino acid residues, namely Asn_159_, Gln_160_, Lys_194_, and Glu_196_ [46]. These results indicated that BMD42-2910 is a promising novel anti-prion compound, which should be investigated further for keeping clinical trials in mind.

## Carbazole derivatives

GJP14 or [2,3,4,9-Tetrahydro-9-[2-hydroxy-3-(1-piperidinyl) propyl]-6-methyl-1 H-carbazol-1-one] has inhibited PrP^Sc^ accumulation (IC_50_ 8.54 µM) in cellular assay by GT+FK cells [mouse neuronal cells (GT1-7) persistently infected with mouse-adapted GSS agent (Fukuoka-1 strain)] [47]. Taking it as a lead compound, various derivatives have been synthesized and screened for anti-prion activity. The SAR revealed the importance of the tricyclic aromatic ring, an amino group at the 3-position, and a hydroxy group at the 2-position of the N-propyl group for displaying anti-prion activity. As a result, the N-ortho-fluorobenzyl analogues (IC_50_ 1.11 µM) was found to be the most effective approximately 8 times stronger than the parent compound [47]. The binding properties of GJP14 were elucidated by surface plasmon resonance (SPR) and NMR spectroscopy, which displayed a ligand-binding pocket at the C-terminal domain of PrP^C^ [48]. Nevertheless, reports on *in vivo* studies of GJP14 on prion-infected models are pending.

## Cyclic tetrapyrroles

Cyclic tetrapyrroles are planar aromatic ring structures with metal ions, defined by peripheral substituent groups of different chemical nature. Porphyrins (deuteroporphyrin IX 2,4-bis-(ethylene glycol) iron(III) (DPG2-Fe3+), meso-tetra (2- N-methylpyridyl)porphine iron(III) (TMPP-Fe^3+^), tetra (4- N,N,N-trimethylanilinium)porphine (Fe-TAP), tetra (4- sulphonatophenyl)porphine (Fe-TSP), haemin) and phthalocyanines belong to this group. Tetrapyrroles have been reported to inhibit PrP^Sc^ oligomerization *in vitro* [49] and increase the survival time *in vivo* [50–52]. SPR revealed the direct interactions of cyclic tetrapyrroles with PrP^C^ or PrP^Sc^ [53], which bind to PrP^C^ by stacking and interrelating through the N-terminal of PrP [54,55]. NMR studies identified residues at the C terminal of helix 3 and in the loop (between residues 160–180) on human PrP as the binding site of Fe(III)-TMPyP (K_D_ 4.52 µM) responsible for inhibition of prion replication (EC_50_ 1.6 µM) [56] and PrP^C^-mediated toxicity in the cell-based assay [57]. Binding of Fe(III)-TMPyP seemed to stabilize the folded PrP conformation both thermodynamically and kinetically [58]. Tetrapyrroles proved as the most potent pharmacological chaperones of PrP^C^ *in vitro* and cell-based assays, but disappointingly, the therapeutic capabilities of porphyrins could not be approved in clinical trials, owing to poor bioavailability and non-specific interactions with the plasma proteins [58].

## Diazo dyes

Congo red (CR) is a conventional diazo dye used for histological studies with binding affinity to fibril proteins rich in β-sheet conformation [59,60]. CR has anti-prion activity and inhibits PrP^Sc^ formations [61] by altering the cellular levels of PrP^C^, stabilizing PrP^Sc^, and preventing the further conversion of PrP^C^ [62–65]. SPR displayed CR binding to human recombinant PrP with K_D_ value of 1.6 µM [66]. A few other related dyes (Sirius Red and other sulphonated dyes) were also reported to block PrP^Sc^ generation *in vitro* [67]. However, despite its strong *in vitro* anti-prion activity, CR is considered potentially toxic (as benzidine derivative is released during catabolism of CR) and displays poor bioavailability *in vivo* [68,69] with high non-specific binding [38]. To increase the bioavailability (by replacing sulphonate of core moiety with carboxylic acid) and decrease toxicity (replacing benzidine), a series of CR analogues were synthesized and screened for anti-prion activity [70]. Different analogues of CR inhibited PrP^Sc^ aggregation in the nano-molar range. The most active compound, 2a, potentially inhibited (EC_50_ 25–50 nM) PrP^Sc^ formation in scrapie mouse brain cells (SMB) [71] and scrapie-infected hamsters [72]. However, no conclusive report is available on its interactions with PrP^C^.

## Diphenylmethane derivatives

Evidence from the *in silico*-based drug screening revealed that GN8, a diphenylmethane derivative, stabilizes PrP^C^ conformation by acting as a chaperone and decreasing PrP^Sc^ levels (IC_50_ ~ 1.35 µM) considerably in a mouse neuronal cell line, prolonging the survival of TSE-infected mice (subcutaneous injection) [73]. SPR confirmed the explicit binding of GN8 to PrP^C^. Most of the residue involved in the binding is located at the C-terminal domain i.e. placed in the S2-A loop, the B-helix, or the B-C loop. The intercalation of the A-S2 loop and B-C loop seems to be crucial for stabilizing PrP^C^ conformation. GN8 connected distant Asp_159_ and Glu_196_ (36 amino acids apart in PrP^C^) by hydrogen bonds, due to which further conformational changes are blocked. The free energy of the PrP^C^–GN8 complex (∆∆H = 6.7 kcal/mol) is considerably lower than that of PrP^C^ alone, which ultimately reduced the chances of the formation of a transition state, stabilizing PrP^C^ conformation [73]. Later, additional GN8 derivatives were synthesized and analysed for anti-prion activity in TSE-infected mouse neuronal cells. SAR indicated better anti-prion activity (IC_50_ 0.51–0.83 μM) of derivatives bearing substituents at the benzylic position [47,74]. Positron emission tomography (PET) images from the ^11^C-labelled compound established that the GN8 derivative can cross BBB and enter the brain [48]. Additionally, the nonclinical safety assessment of GN8 did not show any severe adverse effects at doses sufficient for anti-prion activity [75]. In further studies for better active chaperone, researchers modified GN8 to a medical chaperon (MC: *N,N*'-([cyclohexylmethylene]di-4,1-phenylene)bis(2-[1-pyrrolidinyl]acetamide). MC binding to PrP^C^ reduced the fluctuations in the binding site (amino acid residues 186–196) and increased the instability in other regions (amino acid residues 144–154) of helix A. Moreover, it inhibited PrP^Sc^ oligomerization and favoured its removal in the infected cells. Interestingly, MC exhibited effectiveness in a variety of strains (Fukuoka-1, 22 L, RML) and hosts (mice, macaque), preventing the generation of drug resistance [76]. Due to the encouraging results shown by MC in prion disease, scientists are working towards human clinical trials for prion diseases.

## Diphenylpyrazole derivatives (DPP)

Diphenylpyrazole analogues are potent inhibitors of PrP^Sc^ oligomerization *in vitro*. The best efficient inhibitor, named DPP-1, displayed an IC_50_ value of 0.6 μM and 1.2 μM in SMB and ScN2a cells, respectively [77]. In a SAR study, the methyl group was documented as a significant motif for the improved activity in SMB cells, and not in ScN2a cells; on contrary, the activity dropped in both cell lines when fluorine was replaced by bromine [78]. In ScN2a cells, DPP-1 neither affected PrP^C^ expression nor proteasome activity and also failed to show any effect in a cell-free assay. Nevertheless, it delayed the clinical onset of the disease in mice with no reported toxicity *in vitro* or *in vivo* [77].

Anle138b, 3-(1,3-Benzodioxol-5-yl)-5-(3-bromophenyl)-1 H-pyrazole, inhibited PrP^Sc^ formation in a cell-free protein misfolding cyclic amplification (PMCA) and mice infected with different PrP^Sc^ strains (EC_50_ 7.1 μM for the vCJD; 7.3 μM for the murine RML). Impressively, it also prolonged the survival in late prion infection [79]. Excitingly, Anle138b also inhibited α-synuclein aggregation [80] for its potential therapeutic role in the pathophysiology of Parkinson’s disease (PD). Therefore, Anle138b can be a promising new lead against other protein misfolding diseases. When the mode of action of Anle138b was elucidated using all-atom molecular dynamic stimulations, it interacted with the unorganized protein through its pyrazole moiety, blocking inter-peptide interactions and spontaneous formation of β sheets. These interactions helped in reducing intramolecular hydrogen bonding and remodelling from the allosteric induction. However, another analog, Anle234b, did not affect aggregation in this study [81]. No doubt Anle138b is a non-toxic promising lead compound with the potential for oral administration. Still, it is not a cure as it only delays the disease progression and is not a disease-modifying candidate. A ‘First-in-human study to assess the safety, tolerability, and pharmacokinetics of Anle138b in healthy male and female subjects’ cleared Phase 1 [82]. The outcome of the study indicated that the drug’s half-life is 12 h, and plasma levels significantly exceeded those required for the efficacy. The doses up to 300 mg/day have been considered safe. The company (MODAG GmbH) started a Phase 1b study for PD with Anle138b [81]. The trial will evaluate the well-being, acceptability, and pharmacokinetics of 150 mg and higher daily doses. The study will be completed this year [83].

## dimethyl sulphoxide (DMSO)

DMSO is an aprotic solvent frequently used in laboratories. It has been used in the management of many peripheral amyloidotic diseases since it could block intermediate β-sheet formation [84,85]. DMSO administration has significantly increased the survival time in prion-infected hamsters and decreased PrP^Sc^ accumulation in the brains of infected animals which was later detected in the urine [86]. Although DMSO is unable to solubilize preformed PrP^Sc^ aggregates, it can successfully subdue the PrP^Sc^ accumulation arising during the extraction of prion-infected membranes by detergents [87], suggesting its role as a chemical chaperone [88].

## Ethanolamine

Ethanolamine is a newly discovered anti-prion compound, which effectively reduced PrP^Sc^ levels in prion-infected N2aC24L1-3 cells in a dose-dependent manner. In the same study, oral administration of ethanolamine proved effective against RML prion-infected mice [89]. Ethanolamine neither affected the subcellular localization of PrP^C^ nor disturbed the *in vitro* PrP^C^ to PrP^Sc^ conversion. Ethanolamine is required for phosphatidylethanolamine biosynthesis, a key lipid component of the cellular membranes [90], which in turn facilitated the conversion of PrP^C^ into PrP^Sc^ [91]. Contrarily, in yet another study phosphatidylethanolamine blocked the conversion of PrP^C^ into PrP^Sc^ [92]. Therefore, more investigations should be performed to confirm the role of ethanolamine in prion diseases [89].

## Indole-3-Glyoxylamides

Indole-3-glyoxylamide scaffold is of medicinal significance and is being used in the treatment of atopic dermatitis [93], HIV [94], some cancers [95], and acute coronary syndrome [96]. A series of indole-3-glyoxylamides have been synthesized and assessed for anti-prion activity in SMB cells, where the two most potent compounds, **1** (EC_50_ 1.5 μM) and **2** (EC_50_ 6.4 μM) were identified as anti-prion agents. However, it is interesting to note that indole-3-glyoxylamides did not interact directly with PrP^C^ up to 40 μM [97].

The SAR studies revealed that glyoxylamides derived from primary aromatic amines and bearing a para-substituent are the key requirements for optimum potency in the SMB cell line assay. Based on these results further modifications have been carried out, where indole-3-glyoxylamides unsubstituted at N-1 exhibited higher activity, and there must be no substituent at C-2 for anti-prion activity. The best effective compounds possessed a *p*-amino substituent: *p*-piperidino > *p*-NMe_2_ > *p*-morpholino > *p*-pyrrolyl > *p*-1 *H*-pyrazolyl with IC_50_ in nanomolar range (72 nM >26 nM >9 nM >6 > 1 nM, respectively) [97,98]. Additionally, substitution at C-6 (*R*^3^ = 6-Me, 6-CN or 6-NO_2_) resulted in improved stability, low toxicity, and better EC_50_ values in comparison to the unsubstituted parents [99]. However, no report of *in vivo* studies on prion-infected models is available.

## Lithium (Li)

Lithium is a silvery-white alkali metal with potential neuroprotective activity in the NDDs [100,101]. Furthermore, the role of Li as an anti-prion agent has been assessed in prion-infected cell cultures, where Li reduced PrP^Sc^ aggregation through the autophagy mechanism [102]. Li is reported to block the activation of glycogen synthase kinase 3 (GSK-3), a critical enzyme involved in prion-induced neurodegeneration [103]. Song et al. [104,105] suggested that Li exhibited the neuroprotective effect by restoring survival-associated proteins (RE1 Silencing Transcription Factor: REST & Wingless-related integration site: Wnt), reducing reactive oxygen species (ROS), and repairing synapses. However, it had a narrow therapeutic window (0.5–1.2 mEq/L plasma concentration) and is toxic beyond the above concentration [105]. Administration of Li microemulsion (NP03) in prion-infected mice at a wide range of doses (40 µg/kg/day-16 mg/kg/day) extended the survival of infected animals, even at the lowest dose with a slight reduction in PrP^Sc^ level [106]. The encouraging results of NP03-Li micro emulsion in improving the neuropathology are prominent in prion mouse models, even at late stages of the disease in comparison with Li alone, suggesting its possible use in the chronic treatment of prion disease.

## Phenothiazine derivative

Methylene Blue (MB), a phenothiazine derivative, can bind to PrP^C^ and affect oligomerization kinetics [107] in a study with human, ovine, and murine recombinant PrP. NMR results indicated that MB can bind to Asn_146, 156_; Tyr_160_; Lys_188_; Thr_191,194, 195_; Val_192_, and Gln_215_ residues in helix 1–3 [107]. However, the non-specific binding of MB to other proteins could limit its use [108].

## Sulphated polyanions

***Pentosan polysulfate*** ***(PPS)*** is one of the important drugs in prion disease after quinacrine. The polyanionic compounds interact with N-terminal His, while the planar aromatic compounds form π-stacks and intercalate within Trp side chains [55,109]. The binding is responsible for down-regulating PrP^C^ expression [110] or PrP^C^ internalization [111]. Isothermal titration calorimetry (ITC) showed that PPS has two binding sites on PrP, which can bind to multiple PrP molecules, delaying refolding. Force spectroscopy measurements revealed that PPS stabilizes both native PrP and other incompletely folded intermediates of PrP, as a chaperone [112]. PPS has also displayed its anti-prion potential in cell cultures by inhibiting prion proliferation [113] and prolonging the survival times prophylactically and therapeutically in prion-infected mice [114,115]. It was speculated that PPS may competitively affect the binding of PrP^C^ and PrP^Sc^ by endogenous glycosaminoglycans (GAG) [113] or fragment PrP^Sc^ at the cell surface, like heparin-mimetic compounds [116]. In mouse neuroblast cells overexpressing chicken PrP^C^, PPS can enhance the PrP^C^ internalization and redistribution into late endosomes and/or lysosomes [111], in addition to reducing PrP^C^ levels at 100 µg/ml [117]. On the contrary, PPS decreased the PrP^Sc^ levels promptly in N2a-3 cells (a sub clone of mouse neuroblastoma cell line Neuro 2a), without affecting the intracellular distribution of PrP^C^ up to 10 µg/ml [118]. PPS treatment also inhibited PrP^Sc^ aggregation in a CWD-infected deer-cell model with similar efficacy as reported in RML-infected N2a cells [119]. PPS extended the survival in vCJD subjects although no clinical benefit is reported [120]. The poor BBB permeability of PPS can be improved by intraventricular delivery [115,121], but there was no considerable change in the pathology of treated and untreated groups, unfortunately [122].

***Dextran sulphate*** ***(DS)*** displayed minimal cytotoxicity with powerful anti-prion activity by blocking the conversion of PrP^C^ [123]. In N2a-58 cells, DS specifically inhibited PrP^Sc^ attachment to the membrane and its propagation [124]. Its administration also delayed the onset of the disease in mice [125,126] and hamsters [127] within 2 h of infection, afterwards, it became ineffective [125].

***Cyclodextrans*** are used as additives for various purposes in the food industry [128]. The β-form of cyclodextrin was found to be more efficient (IC_50_ 75 µM) in removing PrP^Sc^ from scrapie-infected neuroblastoma cells than the α-form (IC_50_ 750 µM). The SAR discovered that anti-prion activity is reliant on the size of the cyclodextrin. The structural orientation of glucopyranose units in cyclodextrin creates a hydrophobic cavity, which can encapsulate the hydrophobic moieties [129]. The anti-prion activity of cyclodextrin can be enhanced on sulphation due to its chaperoning function for assisting the proper folding of PrP^C^, and not due to its anionic character [130].

## 2. Repurposed drugs

Even though the drugs are also chemical compounds, they will be discussed under a separate heading. In this section, drugs with United States Food and Drug Administration (US-FDA) approval will be discussed, which are already in the market for the treatment of a range of other diseases with acceptable pharmacokinetics and safety profiles. These drugs have been repurposed to study their efficacy in treating prion diseases (Table 2).

**Table 2.** Repurposed drugs as anti-prion compounds.

Name	Class	Structure	Administration route	Strategy	MOA	BBB permeability/Toxicity	Status	Ref
Celecoxib & its derivatives (AR-12, AR-14)	NSAID; COX-2 inhibitor	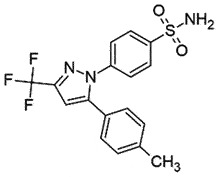	i.p.	Autophagy	Block PrP^Sc^ induced microglia activation & inflammation; reduce PrP^Sc^ accumulation	BBB permeable Non-Toxic	*in vivo,in vitro*	[133–136]
*Chlorpromazine*	Phenothiazine derivative; Antipsychotic drug	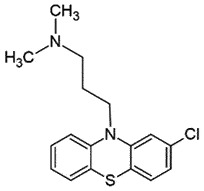	Oral	PrP^C^	Slowly decrease PrP^Sc^ level; Changes subcellular distribution and metabolism of PrP^Sc^	BBB permeable	*in vitro, in vivo*	[53,138–144]
Efavirenz	Non-nucleoside reverse transcriptase inhibitor; Anti-HIV drug	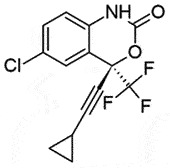	Oral	Cyp_46A1_ activator	Reduce PrP^Sc^ formation	BBB permeable	*in vitro, in vivo*	[145]
Flupirtine	Triaminopyridines; Non-opioid analgesic	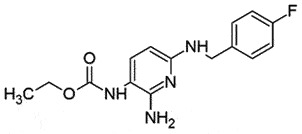	Oral	Neuro-protective; Bcl 2 upregulation	Reduce PrP^Sc^; protect against neurotoxicity and apoptotic cell death induced by PrP fragments	BBB permeable Well tolerated	CJD patients: double-blind study: improved cognition, no effect on disease progression	[146–154]
Glimepiride	Sulphonylurea derivative; Anti-diabetic drug	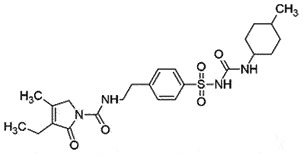		(GPI)-phospholipase C	Reduce expression of PrP^C^; Reduce PrP^Sc^ levels; Anti-inflammatory	Poor BBB ability	*in vitro*	[161–165]
Guanabenz	α2-AR agonist; Antihypertensive drug	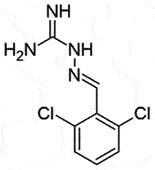	Oral i.p.	PrP^C^	Promote PrP^Sc^ clearance	BBB permeable Non toxic	*in vivo,in vitro,ex vivo*	[156–159]
Imatinib	Tyrosine kinase inhibitor; Anticancer drug	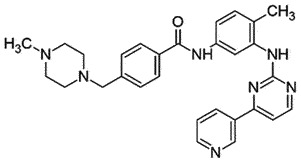	i.p.	Autophagy	Lysosomal clearance of PrP^Sc^	Poor BBB ability	*in vivo,in vitro*	[166–170]
Quinacrine	Acridine derivative; Anti-malarial drug	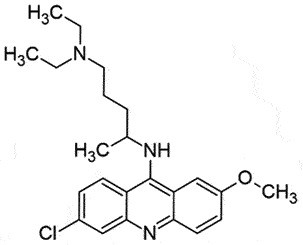	Oral	PrP^C^	Inhibit PrP^Sc^ formation	BBB permeable Non-toxic up to 300 mg/day	Failed Phase II trial on CJD patients	[53,139,140,171–180]
Radotinib	Tyrosine kinase inhibitor; Anticancer drug	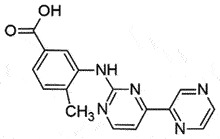		NA	Reduce PrP^Sc^ accumulation	BBB permeable Non-toxic up to 1 g/kg	*in vivo*, patent filed	[184,185]
Simvastatin	HMG-CoA inhibitor; Cholesterol lowering medication	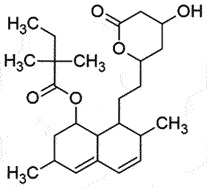	Oral	Inhibit protein prenylation; anti-inflammatory; antioxidant	Reduce PrP^Sc^ levels	BBB permeable	*In vivo*	[186–194]
Tetracycline Doxycycline Amphotericin B	Antibiotics	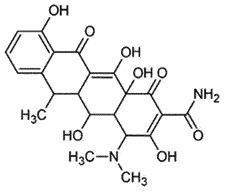	Oral	PrP^C^	Reduce PrP^Sc^ levels	BBB permeable Safe (Tetracycline and Doxycycline) AmB: toxicity issues	*in vitro, in vivo* Doxycycline: Phase II CJD:No effect in late stages of CJD	[196–209]

## Celecoxib

Celecoxib is a FDA-approved non-steroidal anti-inflammatory drug (NSAID) [131] for inhibiting cycloxygenase (COX-2). It can also block PrP^Sc^-induced microglia activation and releases prostaglandin (PGE2) and nitric oxide (NO) [132]. On the other hand, ketoprofen (COX-1 inhibitor) was not able to inhibit microglial activation and the associated reactions caused by PrP^Sc^, suggesting the role of PrP^Sc^-induced inflammation in prion progression [132]. An anticancer agent, celecoxib derivative: AR-12 (OSU-03012), was assessed by FDA as an approved investigational new drug (IND) in phase I of clinical trials for advanced or recurrent solid tumours or lymphoma patients [133]. Abdurahman et al. evaluated the effect of AR-12 and its derivative, and AR-14 revealed anti-prion properties in neuronal and non-neuronal cell culture models [134]. AR-12 showed a significant reduction of PrP^Sc^ in prion-infected ScN2a and ScMEF cells. Prolonged treatment eliminated PrP^Sc^ in a sensitive RT-QuIC analysis, even when the treatment was discontinuation. It has been suggested that both AR-12 and AR-14 can induce autophagy in cells and eradicate infection [134]. Notably, AR-12 can cross BBB successfully [135], which is an essential requirement for an anti-prion drug. Autophagy is the natural cellular degradation and reprocessing pathway, in which the dysfunctional components from the cytoplasm are packed in vesicles and delivered to the lysosomes for degradation. Thus, by enhancing the autophagic flux, PrP^Sc^ lysosomal degradation can also be enhanced, providing a therapeutic approach for prion diseases [136].

## Chlorpromazine *(CPZ)*

CPZ, a phenothiazine derivative, is a US-FDA-approved antipsychotic drug [137], which can cross BBB. CPZ inhibited PrP^Sc^ formation (EC_50_ 3 μM) [138] by binding to human recombinant PrP by acting as a pharmacological chaperone. However, its interactions are weaker than quinacrine (antimalarial drug) [53,139,140]. Unfortunately, CPZ failed to show results in protein misfolding cyclic amplification (PMCA) assay [141] and was unable to inhibit PrP^Sc^ build-up in the cellular assay [142]. It was suggested that CPZ may induce PrP^Sc^ reallocation in late endosomes/lysosomes [143]. CPZ was found to be 10 times less active than quinacrine, which completely inhibited PrP^Sc^ formation at 400 nM [45]. Administration of CPZ prolonged the incubation duration and somewhat reduced the morbidity in mice infected intracerebrally with prions [144].

## Efavirenz (EFV)

EFV is a US-FD- approved oral non-nucleoside reverse transcriptase inhibitor (NNRTI) medication to treat the human immunodeficiency virus (HIV). Importantly, EFV could effectively cross BBB with a significant reduction in Cytochrome P450 Family 46 Subfamily A Member 1 (CYP46A1) levels in prion disease [145]. Therefore, being a CYP46A1 activator, EFV can be used on prion-infected neuronal cells and mice with considerably alleviated PrP^Sc^ proliferation, without affecting PrP^C^ [145]. Hence, EFV might prove to be an effective drug in prion diseases.

## Flupirtine (FLU)

FLU, a triaminopyridines, is a famous, well-tolerated, non-opiate analgesic drug with additional myorelaxant properties [146]. It does not have US-FDA approval but is permitted in Europe [147]. Earlier studies demonstrated that FLU might act as an antagonist of N-methyl-D-aspartate receptor (NMDAR) without binding to PrP^C^ [148], but can interact with NMDARs on neurons and modulate NMDAR-dependent neuronal excitability and excitotoxicity [149]. The binding of PrP^Sc^ to PrP^C^ on the cell surface could initiate a signalling cascade, which leads to the activation of α-amino-3-hydroxy-5-methyl-4-isoxazolepropionic acid receptor (AMPAR) and NMDAR, the inflow of calcium ions, and stimulation of p38 mitogen-activated protein kinase (MAPK): a significant modulator of microglial neuroinflammation. Eventually, the actin cytoskeleton breaks down leading to the degradation of dendritic spines, thus the excitatory neurotransmission decreases [150].

FLU protected the neuronal cells from neurotoxicity, which could be induced by PrP fragmentations and amyloid beta induction *in vitro* [151]. A double-blind study on CJD patients [152] with FLU treatment revealed improved cognition, supporting its ability to cross the BBB [153]. However, FLU failed to show any impact on disease progression or survival period in the same study. The neuroprotective potential of FLU is possibly by its anti-oxidant nature (maintain glutathione levels); defence against glutamate-mediated excitotoxicity and role in apoptotic pathways (increasing anti-apoptotic B-cell lymphoma 2 protein: Bcl-2) [154].

## Guanabenz (GA)

GA: α2-adrenergic receptor (α2-AR) agonist [155] is FDA approved antihypertensive drug in use for a long. In a screening study with anti-prion properties, GA stimulated the clearance of PrP^Sc^ *in vivo* against both yeast and ovine prions. GA is not directly involved in the PrP conversion, instead, it alters the protein folding activity of the ribosome (PFAR) [156]. Moreover, GA is non-toxic and can easily cross BBB [157,158]. To overcome any side effects (anti-hypertensive) of GA in treating prion diseases, removing α2-AR agonist activity would be essential. Hence, based on SAR, modification of the positions of chlorine on benzyl moiety and guanidine was performed. These changes led to derivatives **6** and **7**, which possessed a strong anti-prion activity in comparison to GA (IC_50_ 2.8 ± 1.3 μM;1.1 ± 0.5 μM and 12.5 ± 2.7 μM for **6, 7**, and GA, respectively), independent of α2-AR agonist activity. Like GA, these derivatives exhibited an anti-prion effect by inhibiting PFAR [159]. Therefore, these two GA derivatives would be worth it for the next *in vivo* and clinical studies.

## Glimepiride

Glimepiride is a sulphonylurea derivative, approved by the US FDA for the treatment of type 2 diabetes [160]. The effect of glimepiride was investigated on prion-infected neuronal cells (ScN2a, SMB, and ScGT1 cells). The results indicated that the drug decreased PrP^C^ expression and formation of PrP^Sc^ by activating endogenous glycosylphosphatidylinositol (GPI)-phospholipase C, which reduced the PrP^C^ expressions on the surface of neurons. Additionally, glimepiride also displayed a neuroprotective effect against prion-derived peptide PrP_82-146_ [161]. This peptide is known to induce the activation of cytoplasmic phospholipase A2 (cPLA2) and the production of prostaglandin E2 (PGE2), linked with neuronal injury in prion diseases [162,163]. In addition, glimepiride exerted anti-inflammatory activity by reducing the expression of CD14 (cluster of differentiation 14) and other inflammatory cytokines in the macrophages [164]. From the animal data, glimepiride has been shown to pass through the placenta, but its BBB permeability is low [165].

## Imatinib

Imatinib is a tyrosine kinase inhibitor approved by US FDA as oral chemotherapy medication. Imatinib can induce autophagy [166,167] and accelerate the lysosomal clearance of PrP^Sc^ in prion-infected ScN2a cells and scrapie-infected mouse spleens [166,168]. However, no such effect of clearance was observed in CNS by either intraperitoneal (i.p.) or intracerebroventricular (i.c.) delivery due to its poor BBB penetration [169,170].

## Quinacrine (QC)

QC is an acridine derivative that inhibited the PrP^Sc^ formation (EC_50_ 0.3 µM) effectively in the cell-based assays [171]. US FDA has approved the repurposing QC [172], which is an antimalarial drug with the ability to cross the BBB [173]. As observed by NMR spectroscopy, QC was able to bind to the Tyr_226_, and Gln_227_ of helix- 3 of human recombinant PrP globular domain (amino acid residues 121–230), Tyr_225_ [174] and act as a pharmacological chaperone [140]. QC is also involved in PrP^Sc^ clearance *in vitro* [175]. However, it was unsuccessful with ScGT1 cells (chronically scrapie-infected murine neuronal cells), suggesting its therapeutic potential only after long-term treatments [172]. The cytotoxicity of QC was above 2 μM [45,171] in the cellular assay, and the optimal concentration to prevent PrP^Sc^ formation was ~ 4 μM [172]. Combination therapy using QC with simvastatin or desipramine was found to be more effective than QC alone [176].

The SAR-based studies indicated that (S)- QC exhibits a superior anti-prion activity in comparison to (R)-enantiomer [171]. Another study has shown the importance of nitrogen at position 7 of the tricyclic scaffold and aliphatic side chains for increasing anti-prion activity [138]. Compounds with dimethylaminopropyl side chain (promazine, chlorpromazine, acepromazine) from the ring nitrogen at the 9 position of the tricyclic skeleton exhibited better binding activity (EC_50_ 5 μM). Whereas, promethazine with a dimethylamino-2-methylpropyl side chain was relatively less active (EC_50_ ≈ 8 μM) [138]. Despite the encouraging *in vitro* anti-prion activity of QC, no beneficial effect was observed *in vivo* [172,177]. Moreover, no positive outcome was observed, when QC was assessed in phase 2 clinical trials in CJD patients, rather it led to liver dysfunction [177–180]. Another result from the continuous QC treatment seemed to create drug resistance prions. The transitory build-up of these drug-resistant prions might be responsible for the ineffectiveness of QC *in vivo* [39].

## Radotinib

Radotinib is an oral tyrosine kinase inhibitor that is approved by the Korean FDA for treating Chronic myeloid leukaemia–chronic phase (CML-CP) [181]. It is also considered to treat PD patients [182]. It is structurally similar to Imatinib and demonstrated superior anti-cancer activity in comparison to Imatinib in the pre-clinical studies [183], without toxicity up to 1 g/day in phase I [184]. Radotinib (100 mg/Kg p.o.) has shown promising results in the prion-infected hamster model by decreasing PrP^Sc^ deposition in the brain and extending the survival time in the treatment group [185]. Other details related to the mechanism of action are not available yet.

## Simvastatin

As membrane cholesterol has an important role in the conversion of PrP^C^ to PrP^Sc^ [186,187], cholesterol inhibitors have been explored for their anti-prion activity. Simvastatin is approved by US FDA as a cholesterol-lowering medication. Simvastatin treatment (1 mg/Kg) significantly delayed the progression of prion disease and lengthened the survival times of prion-infected mice [188] without any antagonistic effect of statin in the treatment group [189]. It was proposed that simvastatin could exert its beneficial effects in prion infection through protein prenylation, as statins are already known to inhibit both protein farnesylation and geranylation by inhibiting upstream mevalonate/isoprenoid pathway [190]. Convincing data suggested that the inhibition of isoprenoid synthesis and protein prenylation can reduce Aβ accumulations *in vivo* [191], which will be a better strategic mechanism for facilitating statin-induced neuroprotection [192,193]. Additionally, anti-inflammatory and antioxidant effects will have positive synergies in treating prion diseases [186,194].

## Antibiotics

The resistance to proteinase K digestion is an important characteristic of PrP^Sc^ [27]. Studies revealed that when prions are exposed to antibiotics, they become more prone to enzymatic digestion, accompanied by a reduction in prion infection [195]. As a result, antibiotics are also being reconsidered for brain-wasting diseases, apart from antibiotic activity.

Tetracycline is a well-characterized, safe, and US FDA-approved antibiotic. It protected the cells from prion infection *in vitro* [196,197] and *ex vivo* conditions. Preincubation of prion-infected brain homogenates with tetracycline delayed the onset of clinical symptoms and extended survival in animals. Forloni et al. demonstrated that tetracycline is a potent therapeutic drug for prion diseases, as it targeted fibril formation and stopped neuronal loss and astrocyte proliferation [195]. NMR spectroscopy revealed that tetracycline interacted with amino acid (residues 100–126), a key region in the conformational changes [196]. Docking and molecular simulations suggested a strong interaction of the tetracycline with functional groups of Thr (190–193) in the helix-2 of PrP [198]. Tetracycline might also display indirect neuroprotective effects through antioxidant, anti-inflammatory, and anti-apoptotic mechanisms [199]. A similar molecule, the anthracycline idodoxorubicin, was reported to have an affinity for PrP for reduced prion infection in a rodent model [200,201]. Doxycycline is a second-generation tetracycline antibiotic with better BBB permeability and safety profile [202] and has been reported to reduce PrP^Sc^ levels *in vitro* and extended the survival of prion-infected animals [203]. However, in a randomized, double-blind, placebo-controlled phase 2 clinical trial on CJD, Doxycycline (100 mg/day) was well tolerated without any significant effect in the late stages of CJD [199,204].

Amphotericin B (AmB) is US-FDA approved polyene antibiotic, which also exhibited anti-prion activity [205] but its use is limited because of its toxicity. As a result, derivatives (carboxylic groups replaced by methyl) of AmB were tested for anti-prion potentials and found to be less toxic but equally potent in comparison to AmB [206,207]. While, another derivative of AmB, MS-8209, failed to show a direct effect on PrP^Sc^ [208]. Recently, aminoglycoside G418 (Geneticin) has been reported to interfere with the *de novo* processes involved in earlier stages of prion infection, without affecting the level or localization of PrP [209].

## 3. Natural compounds

Several natural polyphenols have already been reported for their anti-inflammatory, antioxidative, neuroprotective, and pro-autophagic functions [210]. Polyphenols inhibited aggregation of various pathological proteins, like β-amyloid and α-synuclein [211,212] through direct interactions with β-sheets by inhibiting abnormal conformational changes [213,214] or by tilting the metal ion interactions of protein aggregation [215,216]. Apart from natural compounds from plants, other natural compounds with anti-prion potentials, like bile acids, hormones and disaccharides have also been discussed (Table 3).

**Table 3.** Natural compounds as anti-prion agents.

Name	Class	Source	Structure	Administration route	Strategy	MOA	Toxicity/BBB	Status	Ref
Baicalein	Flavonoids	*Scutellaria lateriflora*	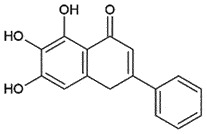	Oral	Neuroprotection	Reduce ROS; Inhibit JNK phosphorylation		*in vitro* *in vivo*	[217–220]
Cannabidiol	Cannabinoid	*Cannabis sativa*	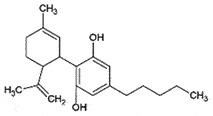		not conclusive	Reduce PrP^Sc^ levels	Can cross BBB Non-toxic	*in vitro*	[224]
Carnosic acid/ Carnosol	Catechol	*Rosmarinus officinalis*	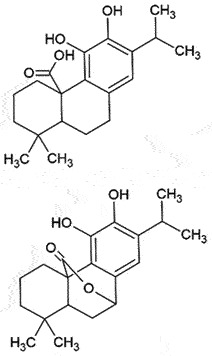		PrP^Sc^	Reduce *de novo* generation of PrP^Sc^ aggregates	Can cross BBB	*in vitro*	[226–228]
Curcumin	Curcuminoid	*Curcuma longa*	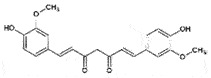	Oral	PrP^C^	Prevent PrP^C^-PrP^Sc^ conversion	Can cross BBB Non-toxic	*in vitro* *in vivo* (ineffective)	[229–234]
Epigallocatechin gallate	Catechins	Green tea	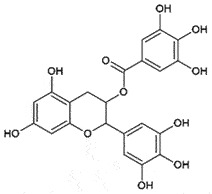		Autophagy	Cellular trafficking of PrP^SC^		*in vitro*	[235–239]
Ginsenosides Rg3	Saponins	Steamed *Ginseng*	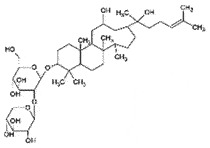		Autophagy	Reduce toxicity of PrP^Sc^		*in vitro*	[240–242]
Hinokitiol	Tropolone derivative	*Cupressus* spp.	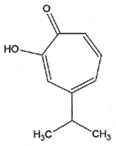		Autophagy	Reduce toxicity of PrP^Sc^		*in vitro*	[243–246]
Melatonin	Neuro-endocrine hormone	Pineal gland	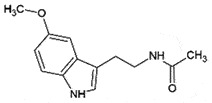		Autophagy	Reduce toxicity of PrP^Sc^		*in vitro* *in vivo*	[249–254]
Polydatin	Resveratrol glycoside	*Polygonum cuspidatum*	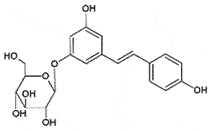		PrP^C^	Prevent PrP^C^-PrP^Sc^ conversion		*in vitro* *in silico*	[257–259]
Resveratrol	Stilbenoid	Grapes/berries	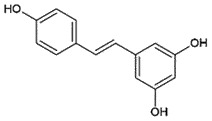		Autophagy	Reduce PrP^Sc^ levels		*in vitro*	[261,262]
Spermine	Polyamine	Eukaryotic cells	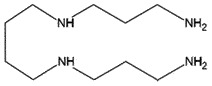		Autophagy	Reduce toxicity of PrP^Sc^ Decrease ROS		*in vitro*	[266]
Tauroursodeoxycholic acid and Ursodeoxycholic acid	Bile acid	Bile	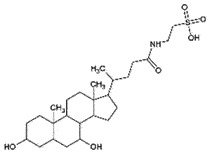 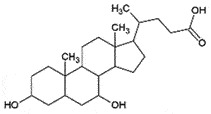		PrP^C^; Chemical chaperone	Reduce PrP^C^-PrP^Sc^ conversion	Can cross BBB Non-toxic	*in vitro* *in vivo*	[221–223]
Trehalose	Disaccharide	Fungi, plants and invertebrates	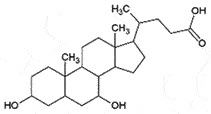	Oral	Autophagy	Reduce PrP^Sc^ accumulation		*in vitro* *in vivo*	[268–271]

## Baicalein

Baicalein, a flavonoid obtained from the mint family herb *Scutellaria lateriflora* (Blue skullcap), is known to be effective in neuroprotection [217]. It has been reported to exert a neuroprotective mechanism and reduce PrP^Sc^ accumulation in SH-SY5Y and SK-N-SH cells *in vitro* [218] by modulating ROS-induced cell death through inhibiting the phosphorylation in the c-Jun N-terminal kinase (JNK) pathway [219]. Moreover, oral administration of *S. lateriflora* (water extract) increased the incubation times in prion-infected mice. It was observed that both baicalein-hydrate and baicalein have almost the same inhibitory effects on prion proliferation and removal of remaining fibrils [220].

## Bile acids

The bile acids, tauroursodeoxycholic acid (TUDCA) and ursodeoxycholic acid (UDCA) are neuroprotective *in vitro, ex vivo*, and *in vivo* by preventing the conversion of PrP^C^ to PrP^Sc^ in ScN2a cells from the reduced neural loss, and increased survival of male mice in prion animal model [221], referring as chemical chaperones [222]. In addition to the promising results, these bile acids are relatively non-toxic, can cross the BBB, and are orally bio-available. Moreover, TUDCA has already been approved by FDA for the treatment of primary biliary cirrhosis [223].

## Cannabidiol (CBD)

CBD, a non-psychoactive compound from *Cannabis* plant, has been reported to inhibit the cerebral accumulation of PrP^Sc^ in prion-infected sheep and mice [224]. Unfortunately, two structurally related cannabinoid analogues, Δ^9^-tetrahydrocannabinol (THC) and endocannabinoid arachidonoyl glycine (AG) proved to be ineffective. The mechanism of action is not conclusive, since CBD did not affect the cellular trafficking of PrP^Sc^ nor it disrupted the pre-existing PrP^Sc^ aggregates. It was suggested that CBD may exhibit neuroprotection by some other mechanism, which reduces PrP^Sc^ levels. Since CBD can cross BBB without drug tolerance from prolonged CBD therapy, it may be a promising anti-prion candidate [224].

## Carnosic acid (CA)

CA, a catechol isolated from *Rosmarinus officinalis* (Rosemary), has been reported for its potential antioxidant, anti-inflammatory, and neuroprotective activities [225]. CA and its metabolite, carnosol (CS), have been shown to exert its potent antioxidant and anti-prion activity in N2a22L and N2a58 cells by reducing *de novo* generation of PrP^Sc^ aggregates, as well as favouring removal of already formed PrP^Sc^ clusters [226]. In addition, CA can cross BBB after oral administration [227], which was also confirmed by Light-BBB computational prediction [228]. Therefore, the direct inhibition of PrP^Sc^ and antioxidant effects could be of specific therapeutic significance, not only in prion diseases but also in others with protein misfolding. Several *in vivo* studies are in progress.

## Curcumin

Curcumin, 1,7-bis (4- hydroxy-3-methoxyphenyl)-1,6-heptadiene-3,5-dione, is the yellow coloured pigment from turmeric (*Curcuma longa*) rhizome. Curcumin has been suggested as an anti-prion compound from partial inhibition of the cell-free conversion of PrP^C^ to PrP^Sc^ and prevention of PrP^Sc^ accumulation in scrapie-infected neuroblastoma cells (IC_50_ ~ 10 nM) [229]. Yet, the *in vivo* studies revealed that dietary administration of curcumin has no substantial effect on scrapie onset in hamsters [229]. In a mouse model, curcumin was able to cross BBB and bind β-amyloid aggregates in the brain [230]. Plasma proteins have been suggested as a carrier of curcumin transport to the brain [231]. Researchers used the circular dichroism (CD) to identify the curcumin binding site on PrP and observed that the binding to the α-helix intermediate blocked the conformational conversion to β-sheet [232]. In a cell-free system, curcumin significantly decreased the fibril formation of the mouse prion protein. In addition, co-incubation with prion amyloid fibrils and curcumin protected the cells against autophagy dysfunction and also decreased the level of ROS in mouse neuroblastoma cells [233]. Since the structure of curcumin has similarities to Congo red, both compounds seemed to compete for binding to the α-helix intermediate. Interestingly, unlike Congo red, curcumin is non-toxic. However, water insolubility (< 0.6 μg/mL) [234] and poor bio-availability are its limitations, which became the restrictions in the clinical trials. Therefore, curcumin in combination with other drugs, the effective drug delivery could be the key in its therapeutic applications.

## Epigallocatechin gallate (EGCG)

EGCG from the green tea extract may be used as a potential therapeutic compound for treating NDDs. In primary neuronal cells, EGCG displayed neuroprotection against prion infection by Bax inhibition, translocation of cytochrome C, activating class III histone deacetylase, and activating autophagy by Sirtulin-1 (sirt-1) induction [235]. The involvement of sirt-1 in prion diseases suggested the maintenance of mitochondrial homoeostasis as a major neuroprotective mechanism [236]. The beneficial effects of sirt-1 overexpression have already been observed in various NDDS [237–239]. Moreover, EGCG promotes PrP^C^ internalization and degradation in lysosomes.

## Ginsenoside-RG3

Ginsenosides Rg3, a saponin from steamed ginseng, is known to have neurogenic potentials [240] by promoting autophagy functions [241]. In a study with ginsenosides Rg3, it protected primary neurons and SK-KSH cells from the induced neurotoxicity of prion peptide (106–126) by promoting autophagy activities [242]. The mechanism of enhanced autophagy functions by ginsenoside-RG3 from prion-induced toxicity is yet to be determined.

## Hinokitiol

Hinokitiol (β-thujaplicin) chemically known as 2-hydroxy-4-isopropylcyclohepta-2,4,6- trien-1-one, is a tropolone derivative from the *Cypressus* trees with neuroprotective pharmacological properties [243,244]. Moon et al. [245] observed that hinokitiol can protect the neuronal cells against PrP^106−126^-induced neurotoxicity through enhanced autophagy functions. Electron microscopy disclosed that hinokitiol boosted the formation of autophagic vacuoles in neurons, where the results advocated the induced autophagy through the hypoxia-inducible factor-1α (HIF-1α) pathway, the key neuroprotective mechanism against PrP-induced toxicity [246].

## Melatonin

Melatonin, a neuroendocrine hormone produced by the pineal gland, is a potent antioxidant molecule [247] linked with various neurodegenerative disorders [248]. Melatonin-induced autophagy exhibited neuroprotective activity in prion-infected cells by modulating mitochondrial functions [249,250]. The pre-treatment with melatonin inhibited the mitochondrial-mediated apoptosis *in vitro* by protecting/modulating the mitochondrial morphology from PrP^106−126^ toxicity by interacting with dynamin-related protein 1 (DRP1) in the mitochondrial fission [251] and optic atrophy 1 (OPA1) in the fusion pathways [252,253]. However, the study of administrating low and high doses of melatonin in animal studies presented the controversial results of stimulating or inhibiting prion activities [254].

## Polydatin

Polydatin, 3,4',5-trihydroxystilbene-3-β-D-glucoside, from roots of Japanese knotweed (*Polygonum cuspidatum*) is a natural precursor of resveratrol [255]. Apart from various pharmaceutical properties [256], polydatin also inhibited amyloid formation [257,258]. In a recent biophysical study, polydatin revealed its anti-prion activity *in vitro* and *in silico* [259] by binding to PrP^C^ and inhibiting the conversion to PrP^Sc^. The detailed structural analysis using molecular dynamics simulations explained that polydatin could suppress the oligomerization by abolishing the unfavourable structural transitions in the β2–α2 loop, α3-helix, and N-terminal amyloidogenic region, favouring α-helical and random coils formation, thereby disabling the prion aggregations and pathogenesis [259].

## Resveratrol

Resveratrol, a polyphenol with antioxidant, anticancer, and neuroprotective properties, is present in a variety of fruits, like grapes and berries [260]. Resveratrol has been reported to decrease the accumulations of PrP^Sc^ (EC_50_ 0.61 μM) in prion-infected SMB-S15 cells in a dose-dependent manner. In addition, one-week treatment with resveratrol significantly reduced the prion infectivity in mice [261], indicating its potential as an anti-prion agent. It is also reported that resveratrol can remove PrP^Sc^ by decreasing sirt-1 (regulates prion protein-induced neuronal cell death) levels in the brain [262]. Structurally, resveratrol interacted with Tyr_128_ by π-π stacking and stabilized PrP^C^ [263] and thus prevented the aggregation. Another neuroprotective potential of resveratrol against prion neurotoxicity is through resveratrol-mediated and enhanced autophagy signals [264].

## Spermine

Spermine. a biologically active polyamine, is essential for cell growth and differentiation. Since the unstable microtubules and influences of atypical protein folding could influence the neurodegenerative processes, the spermine seemed to increase the expressions of proteins of microtubule acetylation (Tubulin alpha1B/1C, Tubulin beta-4B, Tubulin beta-5) for the stabilization of microtubules. Acetylated microtubules play an essential role in trafficking autophagosomes to the lysosomes [265]. Using affinity experiment microtubule, Tubb6 has been identified as a binding partner to spermine, which facilitated the autophagic degradation of PrP^Sc^ in cells [266].

## Trehalose

Trehalose, a non-reducing disaccharide with α, α-1,1-glucosidic bond between two α- glucose units, is present in almost all organisms, except vertebrates, with cellular defence functions against different types of stress conditions [267]. Trehalose can remove protein aggregates, like α-synuclein by activating autophagy in the mammalian target of rapamycin (mTOR) independent pathway [268]. Aguib et al. [269] reported the autophagy induction and simultaneous reduction of PrP^Sc^ in prion-infected cultured cells from the trehalose treatment. In the mice model (intraperitoneally infected with prion), oral administration of trehalose reduced PrP^Sc^ accumulation slightly without affecting the survival of the animals. It is suggested that the trehalose levels in CNS are not high enough to show the desired effects from limited BBB crossing [269]. Trehalose can also protect the prion-infected cells against induced oxidative stress by modifying the subcellular localization of PrP^Sc^ [270]. It is speculated that the neuroprotective effects of trehalose are exhibited through direct or indirect mechanisms, or with involvements of gut microbiota [271].

## 4. Immunotherapy

Immunotherapy comprises both active (vaccine-mediated) and passive (antibody-mediated) immunizations. The proper identification of prion epitope is essential to develop strong specificity and fewer side effects of the therapy [272]. Various encouraging results have been reported from immunotherapy studies with prion-infected animal models.

Immunization with small prion fragments is capable to fit in the furrows of major histocompatibility complex class II (MHC II) by stimulating anti-PrP^C^ immunity for the PrP^Sc^ reduction [273]. However, as the immune system is tolerant to self-antigens, the antibodies gained by immunizations were not frequently effective [274].

As discussed above, PrP^C^ stabilization is quite important to block the conversion of PrP^C^ to PrP^Sc^ hence, a therapy targeting PrP^C^ will have limited neuroprotective effects in prion diseases. However, due to structural differences between PrP^Sc^ and PrP^C^, PrP^Sc^ can also be recognized as an antigen by the immune system and provoke the immune response. This type of immune response has been reported in the terminal stage of prion-infected mice [275]. To develop PrP^Sc-^specific antibody response, the exclusive epitopes on PrP^Sc^ were sought, for which tyrosine-tyrosine-arginine (YYR) repeat motif, a tyrosine-methionine-leucine (YML) motif in β-sheet 1, and the rigid loop (RL) connecting β-sheet 2 to α-helix 2 were identified [276]. The multivalent PrP^Sc^-specific vaccines were prepared using these epitopes and analysed for immunogenicity and safety [277]. The encouraging results of the YYR-based vaccine have been reported in a study on prion-infected deer [278]. Surprisingly, the same vaccine increased the disease onset in elk with the 132 MM genotype and had no effect in elk (132ML genotype), however, the reason was not clear [279].

The binding of PrP^C^-antibodies can block the conversion of PrP^C^ to PrP^Sc^, hence several studies with various antibodies have been carried out extensively.

POM1 and POM6 are globular domain (GD) binding monoclonal antibodies (mAbs) with a distinct biological response as POM1 triggers neurotoxicity while POM6 is neuroprotective. However, it was observed that POM1 toxicity is independent of prion replication [280]. Investigating the link between the PrP^C^ binding of POM1 and the resulting toxicity, ‘H-latch’ (intramolecular hydrogen bond between His_140_ & Arg_208_ of GD) formation was identified as an early reporter of toxicity. Hence designing antibodies that prevent H-latch formation would protect against the damaging effects of prion infection [281].

The simulations studies disclosed that the POM1/PrP interface is more stable compared to POM6/PrP and the binding of antibodies enhances GD flexibility largely (except in *β*_1_-*α*_1_ loop), signifying the contribution of this loop in the pathological alteration [282]. To address the toxicity concern raised by some anti-PrP antibodies, a bi-specific antibody, scPOM-bi, has been designed as a molecular prion tweezer. It is comprised of POM1 and POM2 complementarity determining regions that simultaneously target GD and flexible tail (FT) resulting in strong neuroprotection and might be used as a promising strategy to develop anti-prion immunotherapy [283].

A potent antibody recognizing multiple epitopes in a different region of PrP revealed its inhibitory mechanisms with different effectivity [284]. Monoclonal antibodies (mAbs) significantly restricted the conversion of PrP^C^ in prion-infected cells [285–288] through four epitopes (octarepeat region, residues 90–110, residues 145–160 in helix-1, and residues 210–220 in the C terminal) [289]. Additionally, mAbs directed against regions 90–110 or 145–160 displayed *in vivo* anti-prion activity after intraperitoneal prion infection but not in case of intracerebral infection as these mAbs are inaccessible to BBB.

The anti-PrP antibodies have also been assessed in prion-infected mice models. The 8B4 (recognizing residues 34–52) and 8H4 (recognizing residues 175–185) antibodies were more effective in reducing the prion infection in comparison to 8F9 (recognizing residues 205–233), due to the better affinity for PrP^C^ and PrP^Sc^ [290]. Short-term intraperitoneal treatment with 6D11 also extended the survival in prion-infected mice [291]. It was determined that PrP^C^ and PrP^Sc^ were sequestered by 6D11 from the cell surface and formed complexes (PrP^C^/6D11 and PrP^Sc^/6D11), which would be then exported to lysosomes for degradation [292]. The most efficient antibodies were identified as ICSM 18 (recognizing residues 146–59) and ICSM 35 (recognizing residues 91–110), which increased the survival time considerably in mice, but unfortunately proved ineffective at later stages of the infection or when the mice were infected through the intracerebral route [293], suggesting that these antibodies could not stop the disease progression in CNS, owing to BBB impermeability.

Recent important progress in antibody treatment is the development and clinical efficacy of a humanized anti-PrP^C^ monoclonal antibody (an IgG_4_κ isotype; PRN100), which increased survival in prion-infected mice [293]. PRN100 has been developed by researchers at the Medical Research Council (MRC) Prion Unit, University College London (UCL), after a battery of mouse monoclonal antibodies developed against human PrP [294] for their binding and stabilizing PrP^C^ properties. In a recent clinical study, six CJD patients received PRN100 intravenously every 2 weeks till death or withdrawal from the programme. Through i.v. administration, PRN100 was able to reach the target CSF drug concentration (50 nM), and additionally, it was well tolerated without any adverse effects for up to 8 months [14]. After such promising results, PRN100 will be evaluated for Phase-II trials.

## 5. Clinical trials

Anle138b clinical study was conducted in 2020 with 68 healthy male and female subjects and passed Phase 1 evaluation of safety, tolerability, and pharmacokinetics [82]. Adverse events were considered to be mild and patients fully recovered during this study. In the future, longer and more large-scale evaluations could be conducted to access the full safety profile of Anle138b. Since Anle138b was initially started for the multiple system atrophy treatment and previously hampered α-synuclein aggregation, a Phase 1b trial for 48 patients with PD was initiated in 2020 [81,83]. The trial will assess Anle138b administrated orally at a daily dose of 150 mg and higher for 7 days in PD patients including CSF tests and motor function improvement and is expected to conclude in 2022.

QC has been utilized as an antimalarial drug [173] and also approved as a repurposing anticancer agent [172]. Anaplastic anaemia is the most harmful side effect of QC, which occurs in a lower percentage (0.003%) of patients at high dosages than indicated [295]. A dosage of 300 mg has been reported to be well tolerated in recent clinical trials on QC, but no improvement in survival in CJD patients was observed [177–180]. QC was tolerated at a daily dose of 300 mg but did not affect prion disease in this preference clinical trial [177]. Oral administration of QC for 2 months did not prolong the survival of sCJD patients [178]. Despite the negative outcome, previous clinical trials for CJD showed that proper randomized controlled trials could be conducted in rare, fast progressive, and fatal neuronal disorders.

PPS in humans does not have a proven safe or effective dose regimen. Bone et al. showed that PPS was tolerated up to 110 μg/kg/day doses for 6 months [120]. From two studies, PPS was treated in sporadic, iatrogenic, inherited, and vCJD patients and prolonged mean survival of patients than natural history prion patients with the reduction of abnormal prion protein in the brain [296]. However, treatment with PPS in other studies did not achieve clinical improvement [121], so further studies are needed to clarify the effectiveness of PPS against prion diseases.

FLU was permitted in Europe and Asian countries [147], but later in March 2018, it was withdrawn from the European market. FLU, the first randomized research of a CJD treatment, revealed no effectiveness in survival expansion but did potentially delay cognitive deterioration [152]. Positive results of FLU on cognitive improvement may imply therapeutic benefits in other neurodegenerative disorders.

In a study with a small number of CJD patients, the treatment of doxycycline in the early stage of the disease revealed slightly positive effects [204], but no significant effects were observed in a randomized trial [199]. A doxycycline trial for FFI patients with Asp_178_Asn mutation in the *PRNP* gene is under progress [297].

## Conclusion and future perspectives

Various classes of compounds have been assessed for anti-prion activity *in vitro* and *in vivo*. Different scaffolds displayed anti-prion properties by either direct or indirect interactions with PrP^C^, stabilizing PrP^Sc^, reducing PrP^Sc^ accumulation, or by other neuroprotective mechanisms. Membrane-bound PrP^C^ are largely present in the lipid rafts, consequently, the drugs for inhibiting cholesterol biosynthesis or disturbing raft membranes can redistribute surface PrP^C^, and in that way can reduce accumulation or increase the clearance of PrP^Sc^.

Apart from various strategies that target Prp^C^ or PrP^Sc^, a novel non-PrP targeted drug strategy emerged as an effective approach to treat prion disease [298]. Upregulation of SerpinA3/SerpinA3n has been associated with NDDs, including prion disease [299] as a serine protease inhibitor, it retards the protease activity responsible for clearing prion protein aggregates. With this idea, a small molecule serpin inhibitor (ARN1468) has been designed which effectively reduced prion load in scrapie’s infected ScN2a RML and ScN2a 22 L cell lines. Unfortunately, the low bioavailability of the compound limited the *in vivo* studies [298]. Analogues of ARN1468 with enhanced bioavailability could be promising strategies in treating prion disease.

The ubiquitin-proteasome system (UPS) with an important function in degrading intracellular proteins in eukaryotes seemed to be closely involved in the clearance of protein aggregates. In certain neurodegenerative disorders, this activity has been compromised and led to the accumulation of toxic aggregates of misfolded proteins [300]. Thus, augmenting UPS activity by increasing ubiquitin [301], overexpressing specifically related genes [302], or by inhibiting p38 mitogen-activated protein kinase (MAPK) pathways could also be good candidates for therapies through UPS activations [303].

Chaperones have a key role in the re-folding of cellular protein thereby maintaining proteostasis. However, the proteostasis capacity decreases with age and hence enhances the manifestation of NDDs. Additionally, since the pharmaceutical chaperones usually have a short-half life, maintaining their high concentrations may prevent the protein misfolding as well as reduce the toxicity of protein aggregates. On the other hand, hydrophobic chaperones are less toxic and more suitable for the therapeutic purpose [222]. Literature also suggested the important functions of autophagy in prion disease, where much evidence of autophagosomes in the infected brains would degrade protein aggregates [136]. Yet, the enhanced activity of autophagosomes by potential compounds may not be adequate to eradicate prion infection through induced/enhanced autophagy-flux. Many natural compounds and tyrosine kinase inhibitors could work through this mechanism in ameliorating prion pathology. Due to abnormal protein aggregation, a dramatic increase in oxidative stress and loss of antioxidant defence has been observed in prion diseases. Therefore, compounds reducing oxidative stress could be also beneficial in reducing neurotoxicity and neuroinflammation associated with the disease.

Lately, the encouraging results of a monoclonal antibody (PRN100) in CJD patients could pave the way for new anti-prion agents and will be helpful in the treatments of other NDDs. The success story did not end here, as another promising molecule MC and GN8 would be ready for human clinical trials on prion diseases, while Anle138b was in Phase 1b for PD patients.

As mentioned earlier, quinacrine, pentosan polysulfate, flupirtine, and doxycycline failed in clinical trials on CJD patients. The ineffectiveness of most of the molecules tested was either due to the inability to cross BBB, toxicity, or transitory accumulations of drug-resistant prions. Hence, it would be incredibly important to learn from the backstory. By understanding what was already investigated, what would be the mechanism of drug action, and why the clinical trials failed, we could comprehend prion diseases better and chalk out the right direction for more effective treatments. Lastly, targeting more than one pathway involved in prion diseases may provide synergistic benefits.
